# Finding of an Unusually Colored Sporocyst of the Genus *Leucochloridium* in a *Succinea putris* Snail

**DOI:** 10.1134/S0012496623700473

**Published:** 2023-10-13

**Authors:** R. R. Usmanova, E. E. Prokhorova

**Affiliations:** Laboratory of Experimental Zoology, Herzen State Pedagogical University, 191186 St. Petersburg, Russia

**Keywords:** trematodes, *Leucochloridium*, rDNA, genotyping, coloration, *Succinea putris*

## Abstract

A *Leucochloridium* sp. Carus, 1835 sporocyst with a mature colored broodsac was found in a *Succinea putris* L., 1758 snail in the Boksitogorsk district of Leningrad Region (Russia). The pigmentation of the sporocyst’s broodsac was different from those of other *Leucochloridium* Carus, 1835 species previously described for Europe. The obtained sporocyst is most similar by the coloration of its broodsac to the trematodes of the same genus from Japan. The genotyping of the investigated sporocyst by the rDNA gene fragments (complete ITS1, ITS2, 5.8S and partial 18S and 28S sequences) was conducted. Genetic distances between the obtained sporocyst and the previously described species of the genus *Leucochloridium* were higher than intraspecific ones in the most cases. These data and the data of morphological analysis imply that the investigated sporocyst belongs to a separate species of the *Leucochloridium* genus, previously not described in the European region.

As a rule, trematodes are identified based on the marita and cercaria stages. However, parthenites of trematodes of the genus *Leucochloridium* Carus, 1835 (Trematoda, Leucochloridiidae) have a morphology that allows species identification based on sporocysts. Sporocysts of different species of this genus differ in shape and pigmentation of mature broodsacs (see [1]). Validity of these morphological traits was confirmed by molecular genetic analysis [2–4].

In the course of our long-term monitoring of trematode infection in *Succinea putris* L., 1758 (Gastropoda, Succineidae) carried out since 2008 in the European part of Russia and in Belarus, we have detected three species of the genus *Leucochloridium*: *L. paradoxum* Carus, 1835, *L. vogtianum* Baudon, 1881, and *L. perturbatum* Pojmanska, 1969*.*

The most common findings were sporocysts of *L. paradoxum* characterized by green broodsac pigmentation. The pigmentation pattern exhibited certain variability [5].

In 2017, in the Boksitogorsk district of Leningrad Region, we found a sporocyst with a mature broodsac that also had green pigmentation, but its pattern differed significantly from those described for *L. paradoxum* from Europe by other authors and in our previous works [2, 4, 6, 7]. At the same time, the broodsac pigmentation resembled the pattern described for *Leucochloridium* spp. from Japan [8, 9]. To determine the species affiliation of the discovered sporocyst, we performed a molecular genetic analysis.

## MATERIALS AND METHODS

*Succinea putris* snails infected with sporocysts of *Leucochloridium* sp. and *L. paradoxum* were found in Leningrad Region (59°28′36.6 N, 33°48′21.0 E). The sporocyst of *L. paradoxum* was identified previously based on the morphological traits, as well as by genotyping of a mitochondrial gene fragment of cytochrome *c* oxidase subunit I (GenBank MZ676717.2) [10]. Broodsac pigmentation patterns were analyzed using a Leica M165C stereomicroscope and a Leica DFC290 camera. The *Leucochloridium* sp. sporocyst isolated from the snail was stored at –80°C. DNA was isolated using a DNA-sorb-C-M kit (cat. no. K1-6-50-Mod; AmpliSens, Russia) according to the manufacturer’s instructions. The sporocyst was genotyped based on the rDNA fragment including the complete ITS1, ITS2, and 5.8S rRNA gene sequences, as well as partial 18S and 28S rRNA gene sequences. PCR was performed with three pairs of specific primers: Br/digl1 [11], dig12/1500R [12], and RiboN_F/L2 [3]. The RiboN_F primer was designed using the Primer 3 software [13] based on the nucleotide sequence of *L. paradoxum* (GenBank, KP938187.1). PCR was carried out using *Taq* DNA polymerase (cat. no. EP0401; Thermo Fisher Scientific, Lithuania) with annealing temperatures of 54.7, 56.0*,* and 64.0°C, respectively, according to the protocol described previously [14]. The PCR products were isolated and purified from an agarose gel using a Wizard® SV Gel and PCR Clean-Up System (cat. no. A9281; Promega, United States) according to the manufacturer’s instructions. Sequencing by Sanger was carried out on a commercial basis by Syntol (Russia).

Analysis, assembly and alignment of the sequences were performed using BioEdit v. 7.2.5 [15] and MEGA v. 10.2.4 [16].

Genetic distances (p-distances) were calculated with MEGA v. 10.2.4 [16]. The mathematical model used to calculate genetic distances for phylogeny reconstruction was selected based on the Akaike information criterion and the Bayesian information criterion with jModelTest v. 2.1.7 software [17]. Phylogeny reconstruction based on the ITS1-5.8S-ITS2 rDNA region used a Jukes–Cantor model with two parameters [18]. Phylogeny reconstruction using the maximum likelihood method (ML) was carried out with MEGA v. 7.0 software [19]. Bootstrap support values for the ML trees were calculated based on 1000 replicates [20]. Bayesian inference (BI) was calculated with BEAST v. 2.5 [21] by simultaneously launching four chains of 10^7^ generations and selecting every 1000th generation. Significance of the BI tree topology was estimated based on the a posteriori probability values. The phylogenetic tress generated with TreeAnnotator 1.7.5 were visualized using FigTree 1.4.0 (http://tree.bio.ed.ac.uk/software/figtree/). GenBank accession numbers of the sequences used are indicated next to the tree branches. Intraspecific genetic distances were calculated using the GenBank sequences of *L. paradoxum* (KP938187.1, LC466801.1, MH101511.1, KP903688.1, JF346883.1, JF274482.1, KP903686.1, KP903685.1, KP903686.1, JN639012.1, MK377352.1), *L. perturbatum* (LC466802.1, KP938186.1, KP903687.1, MK377349.1, MK377351.1, JF331664.1), and *L. vogtianum* (KU351661.1, KP903691.1, KP903689.1, KP903690.1).

## RESULTS AND DISCUSSION

**Fig. 1.**
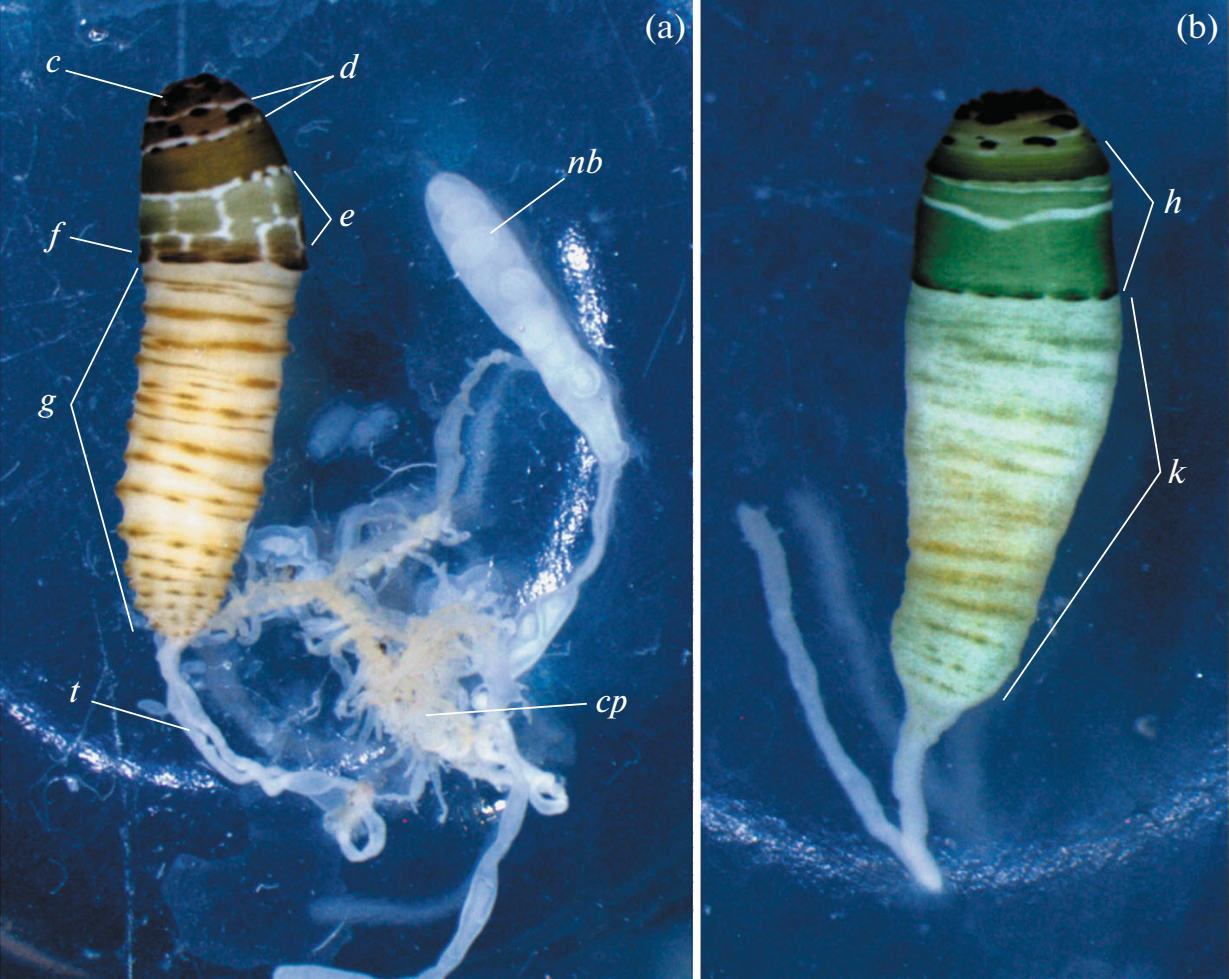
Sporocysts of *Leucochloridium* trematodes. (a) Sporocyst of *Leucochloridium* sp. found in a *Succinea putris* snail: *c*, distal area of brown color; *d*, white transverse bands; *e*, white area with mosaically arranged green square blocks;  *f*, brown dotted band; *g*, yellow-colored part of the broodsac with peculiar lightly brown folds; *cp*, central part of sporocyst’s stolon; *nb*, non-colored broodsac; *t*, tube. *, colored broodsac. (b) Broodsac of *L. paradoxum*: *h*, green area; *k*, proximal part of the broodsac.

**Morphological analysis.** The principal morphological traits used for species identification of *Leucochloridium* trematode sporocysts is the shape and coloration of mature broodsacs [2, 4, 6]. The sporocyst concerned featured an immature (uncolored) and a mature (pigmented) broodsacs (Fig. 1a).

The distal part of the mature broodsac had brown pigmentation. In the next zone, dark protuberant spots and two transverse white bands were seen on the brown-green background. Below, there was an area with a mosaic pattern of green squares on a white background. Adjacent to this area was a distinct discontinuous band of brown color. The rest of the broodsac had yellow pigmentation, and the sporocyst covers formed here circular folds colored with interrupted brown stripes.

Previously, sporocysts with broodsacs featuring this type of pigmentation were not found in Europe. The most similar pigmentation pattern was described for sporocysts of *L. paradoxum* [2, 4–7, 22, 23], which are colored predominantly green and brown (Fig. 1b). However, the broodsacs of *L. paradoxum* sporocysts from Europe do not exhibit a brown band next to the green zone (Fig. 1b). Furthermore, the proximal part of *L. paradoxum* broodsacs has a different pigmentation pattern (continuous bands on a light background) and features no circular folds.

Among the known descriptions of sporocyst broodsacs of the genus *Leucochloridium*, the pigmentation pattern of the sporocyst discovered in Boksitogorsky district resembled most the sporocysts found in Japan and identified as *Leucochloridium* sp. [9] and *L. paradoxum* [8].

**Molecular genetic analysis.** To investigate the discovered *Leucochloridium* sp. sporocyst, we obtained a 1548 bp fragment of rDNA (GenBank, OP709269) that included the complete nucleotide sequences of ITS1, ITS2, and the 5.8S rRNA gene, as well as partial sequences of the 18S and 28S rRNA genes.

**Fig. 2.**
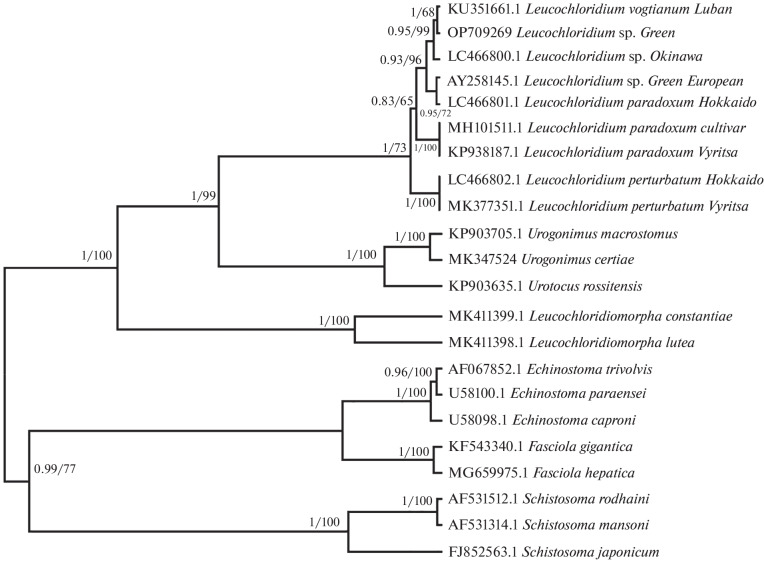
The Bayesian phylogenetic reconstruction based on nucleotide sequences of ITS1-5.8S-ITS2 rDNA (1279 bp) with the use of Jukes–Cantor model. The tree obtained by the maximum likelihood method had the same topology. Numbers at the the branch nodes indicate posterior probabilities for BI and bootstrap support percentages for 1000 replicates for ML. GenBank numbers of the sequences are shown.

In the phylogenetic tree based on the ITS1-5.8S-ITS2 rDNA fragment (1146 bp), species of the family Leucochloridiidae formed a single clade (Fig. 2). Species of the genera *Urogonimus* Monticell, 1888, *Urotocus* Looss, 1899, and *Leucochloridium* formed separate branches. The sporocyst under study was in the same group as *L. paradoxum* from Japan, *Leucochloridium* sp. from Okinawa, and the green sporocyst from Europe; its closest neighbor was *L. vogtianum.*

The genetic distances between the sequences used (Fig. 2) are presented in Table 1. To compare the differences among specimens, we calculated mean intraspecific genetic distances for species of the genus *Leucochloridium* (Table 2). The sequence utilized for this analysis was shorter than the one used for the phylogeny reconstruction (Fig. 2): this way, a larger number of sequences could be included in the alignment.

**Table 1.** Genetic distances (p-distance) between *Leucochloridium* trematodes by the ITS1-5.8S-ITS2 rDNA fragment (1146 bp)

	Species	*Leucochloridium* sp. OP709269	1	2	3	4	5
1	*Leucochloridium* sp*.* Green European AY258145.1	0.01438					
2	*Leucochloridium* *paradoxum* Europe KP938187.1, MH101511.1	0.04167	0.03820				
3	*Leucochloridium paradoxum* Hokkaido LC466801.1	0.01530	0.00278	0.03989			
4	*Leucochloridium* *vogtianum* KU351661.1	0.00668	0.01918	0.04840	0.02199		
5	*Leucochloridium* sp. Okinawa LC466800.1	0.00769	0.01157	0.04086	0.01420	0.01442	
6	*Leucochloridium perturbatum* LC466802.1	0.03693	0.03122	0.03400	0.03152	0.04369	0.03700

**Table 2.** Intraspecific and interspecific genetic distances (p-distance) for *Leucochloridium* trematodes by the ITS1-5.8S-ITS2 rDNA fragment (946 bp)

Parameter	*L. paradoxum*	*L. perturbatum*	*L. vogtianum*
Mean intraspecific p-distance	0.01987	0.00762	0.00059
Mean intraspecific p-distance for the genus *Leucochloridium*	0.00936		
Interspecific p-distance to *L. paradoxum*		0.03329	0.02148
Mean interspecific p-distance	0.02878		
p-distance to *Leucochloridium* sp*.* OP709269	0.02118	0.03127	0.00029
Mean p-distance between *Leucochloridium* sp. OP709269 and other *Leucochloridium* species	0.01758		

The mean genetic distances between the sporocyst studied and other *Leucochloridium* species based on the ITS1-5.8S-ITS2 rDNA fragment were greater than the mean intraspecific distances for *Leucochloridium* members (Table 2). The only exceptions were *L. vogtianum* and *Leucochloridium* sp. from Okinawa: the genetic distances between our specimen and each of these trematodes were smaller than the mean intraspecific distance. The sporocyst studied had the largest distances from *L. perturbatum* and *L. paradoxum*; they were greater than the mean interspecific distance in this genus.

We also obtained a short sequence (339 bp) representing 28S rDNA. This genome fragment is fairly conservative: the genetic distances by this marker between different species of the genus *Leucochloridium* do not exceed 0.009 [8]. Our analysis revealed a small number of substitutions (one to four) within this sequence fragment (Table 3). The sporocyst under study had the most difference from *L. vogtianum* and the least difference from *L. paradoxum* from Japan. This result disagrees with the data obtained for the fragment including internal transcribed spacers and 5.8S rDNA, which was probably due to the conservativity of the 28S fragment studied, as well as to its short length.

**Table 3.** Number of differences between the 28S rDNA fragment sequences (339 bp) of *Leucochloridium* trematodes

	Species	*Leucochloridium* sp. OP709269	1	2	3	4
1	*Leucochloridium paradoxum* Europe KP938187.1, MH101511.1	1				
2	*Leucochloridium paradoxum* Hokkaido LC466801.1	–	1			
3	*Leucochloridium perturbatum* LC466799.1	1	–	1		
4	*Leucochloridium passeri* ON219927.1	1	–	1	–	
5	*Leucochloridium vogtianum* KU351661	3	4	3	4	4

Line indicates the absence of differences.

The results of phylogenetic analysis based on the ITS1-5.8S-ITS2 rDNA fragment suggest that the closest relations of the found sporocyst are *Leucochloridium* sp*.* from Okinawa [8] and *L. vogtianum*. However, the broodsacs of these sporocysts differ significantly in their morphology (shape and/or coloration) from the broodsac of the sporocyst studied. The parthenites of *L. paradoxum* from Japan [8] resembled the one found in our work in broodsac pigmentation, but in the phylogenetic tree it was more distant than sporocysts with dissimilar broodsac coloration.

The data obtained by morphological and molecular genetic analysis of the found sporocyst may indicate either that some of the known species has a high degree of morphological variation or that this sporocyst represents a separate *Leucochloridium* species that has not been described previously in the European region. In our opinion, the latter possibility is more likely, because all genotyped species of the genus *Leucochloridium* had a high level of intraspecies rDNA conservativeness [3, 8, 24] and no cases of basically different coloration in sporocysts of the same species have been described in available publications.
